# Evaluating the impact of a BOPPPS and scenario-based simulation intervention on the competency of junior circulating nurses: a quasi-experimental study

**DOI:** 10.1186/s12909-026-08712-y

**Published:** 2026-02-02

**Authors:** Li Mu, Cheng Wang

**Affiliations:** Operating Room, Peking University First Hospital, No. 7 Xishiku Street, Xicheng District, Beijing, 100034 China

**Keywords:** Models, Educational, Operating Room Nursing, Simulation Training

## Abstract

**Background:**

While enhancing perioperative nursing competency is crucial for improving surgical quality and patient safety, conventional apprenticeship-based training models often fall short in addressing the systematic clinical training needs of junior circulating nurses. This study aims to bridge this gap by innovatively integrating scenario-based simulation with the structured BOPPPS teaching model to enhance training efficacy and clinical performance.

**Objective:**

The aim of this study was to evaluate the effectiveness of integrating the BOPPPS (bridge-in, objective, pre-assessment, participatory learning, post-assessment, summary) teaching model with scenario-based simulation in the training of junior nurses in operating room circulation roles.

**Methods:**

This study employed a quasi-experimental design utilizing a pre-post self-controlled approach.From September to October 2023, 20 junior circulating nurses from a single hospital were randomly assigned to five groups. A structured teaching framework based on the BOPPPS model was implemented, with scenario-based simulation incorporated into the participatory learning component to support training in circulation responsibilities. Each group was randomly assigned one clinical case for simulation. Pre- and post-training assessments were conducted to evaluate the quality of circulation work, work execution rate, incidence of adverse events, and training satisfaction.

**Results:**

Following the training, the quality of circulating work significantly increased from 71.6 ± 6.37 points to 85.05 ± 3.63 points (t = 30.19, *p* < .05), and the work completion rate improved from 69.95% to 82.55% (t = 30.20, *p* < .05). Additionally, the incidence of adverse events decreased markedly, and the overall satisfaction of nurses with the training program reached 96.11%.

**Conclusion:**

This study integrated the BOPPPS teaching model with scenario simulation training, resulting in a significant improvement of 13.45 points (*p* < .05) in the quality of circulating work among junior operating room nurses, along with a 12.6% increase in task completion rate. It is recommended to further optimize the training process by refining the interpretation of learning objectives, dynamically adjusting the difficulty of simulation cases, incorporating self-assessment of job competency, and tracking medium- to long-term outcomes. Additionally, the ADDIE model could be applied to support continuous improvement of the training system.

**Supplementary Information:**

The online version contains supplementary material available at 10.1186/s12909-026-08712-y.

## Introduction

Operating rooms serve as critical platform departments within hospitals, where the quality of nursing care directly impacts the prognosis and safety of surgical patients. As essential members of the surgical team, circulating nurses undertake multiple responsibilities, including comprehensive intraoperative patient management, provision and maintenance of instruments and equipment, team coordination, and emergency response. Their professional competence is a pivotal factor in ensuring smooth surgical procedures and patient safety. However, junior circulating nurses (typically with 3 to 5 years of experience), while having moved beyond the novice stage, still possess relatively limited clinical experience. When confronted with complex surgeries, rare cases, or sudden emergencies, their abilities in knowledge integration, clinical decision-making, and adaptive response are often severely tested. This gap between existing capabilities and clinical demands may lead to elevated levels of job-related stress and anxiety among these nurses. Consequently, this can potentially compromise the precision of nursing interventions, hinder seamless teamwork, and ultimately pose a threat to patient safety.

In 2023, the National Health Commission of China promulgated the Action Plan for Comprehensively Improving Medical Quality, which explicitly prioritizes the enhancement of surgical quality and the reduction of associated complications. This directive imposes unprecedented, elevated demands for the professionalization, standardization, and homogenization of operating room nursing practices.Traditional training for novice nurses predominantly relies on an apprenticeship model, characterized by the transmission of experiential knowledge. While this approach offers certain context-specific relevance, it frequently lacks systematic instructional design. Training content is often contingent upon the individual mentor’s experience, typically follows short-term learning cycles, and is hampered by insufficient feedback mechanisms. Consequently, this model proves inadequate for comprehensively and efficiently cultivating the critical thinking and integrated management competencies essential for nurses to perform effectively within complex, dynamic clinical environments.Therefore, the exploration and development of a more scientific, systematic, and efficacious training paradigm has emerged as a pressing and urgent issue within the realms of operating room nursing management and professional development.

In response to the national strategic mandate for comprehensively enhancing surgical quality and to address the inherent structural deficiencies of the traditional “apprenticeship-based model” for operating room nurse training—such as content randomization, delayed feedback, and fragmented assessment, alongside potential issues like ambiguous objectives and superficial reflection when employing scenario-based simulation in isolation [1]—the core direction of pedagogical reform lies in achieving deep complementarity between systematic instructional design and high-fidelity clinical practice. This necessitates the construction of an integrated training paradigm that transcends the mere superposition of individual methods. Its key is to employ rigorous pedagogical theory as a bridge, infusing the closed-loop, controllable, and structured strengths of the BOPPPS model into the dynamic, immersive learning experience of scenario-based simulation. Consequently, this integration ensures that each simulated training session serves explicit, measurable competency outcomes and promotes long-term knowledge consolidation and transference to clinical practice.

To achieve the aforementioned deep integration, a clear conceptual framework is indispensable. The Jeffries Simulation Theory [2] provides an ideal intermediary for this purpose. Developed by the National League for Nursing (NLN), this theoretical framework for simulation-based education in nursing synthesizes perspectives and evidence from nursing, medicine, and related disciplines, integrating theoretical insights and practical literature. Grounded in experiential learning theory, it has undergone extensive theoretical development and empirical validation by numerous education and healthcare experts. Widely adopted in countries such as the United Kingdom and the United States, it is applied to guide and evaluate instructional design, teaching practices, learning outcomes, and related influencing factors. Its scientific rigor and practical utility have been substantiated [3, 4], establishing it as a mature simulation pedagogy theory [5]. This framework ensures that the entire training process—from stimulating learner motivation to consolidating knowledge—is underpinned by a scientifically designed structure that is goal-oriented, provides immediate feedback, and tightly integrates theory with practice. It effectively cultivates the high-order competencies required in complex and dynamic environments, such as clinical reasoning, emergency management, and teamwork [6, 7], serving as a vital pathway for advancing the operating room nursing profession towards standardized, high-quality development.

Existing empirical studies provide preliminary evidence for the effectiveness of the integrated BOPPPS and scenario-based simulation model in nursing education. The application of this model by Ma et al. [8] in bladder cancer care instruction demonstrated its capacity to concurrently enhance nursing interns’ theoretical knowledge, procedural skills, and core clinical thinking abilities such as critical thinking, alongside achieving higher levels of teaching satisfaction. The research by Cai et al. [9] further indicates that this integrated model also shows advantages in cultivating “soft skills” like humanistic care and empathy, suggesting its pedagogical benefits extend beyond the purely technical domain. The implementation by Wang et al. [10] in an emergency department setting revealed the model’s reinforcing effect on emergency response skills and teamwork capabilities in acute and critical care scenarios. Collectively, these cross-disciplinary studies validate a central thesis: the systematic instructional design provided by BOPPPS—featuring clear objectives, pre- and post-assessment, and structured summarization—can effectively organize and deepen the learning experience within simulated scenarios, thereby generating synergistic learning outcomes across the knowledge, skill, and attitude dimensions. However, these studies predominantly focus on specific disease management or isolated skill training. While their successful experiences offer valuable insights, they essentially represent optimizations of discrete competency modules.

A current limitation in research lies in the fact that this integrated model has not yet been systematically applied to the training of comprehensive, high-intensity roles such as the operating room circulating nurse, which demands the real-time integration of knowledge, technique, situational judgment, and team communication. The complexity and unpredictability of the operating room environment necessitate that training must transcend the enhancement of isolated skills and instead focus on cultivating comprehensive competencies in clinical decision-making, priority management, and systematic problem-solving within dynamic workflows. The direction for deepening future research involves constructing a training system grounded in the BOPPPS instructional framework and centered on high-fidelity, panoramic operating room simulation. This system should prioritize exploring how the “Pre-assessment” phase of BOPPPS can be utilized in depth for customized pre-simulation preparation, how “Participatory Learning” can be transformed into immersive drills of the circulating nurse’s full-range responsibilities, and how “Post-assessment and Summary” can be leveraged for behavioral evidence-based, in-depth debriefing. Consequently, this approach aims to fundamentally shift learning from merely “experiencing” isolated events toward “mastering” transferable core competencies and cognitive models, thereby addressing the critical training challenges in transitioning from novice to proficient practitioner.

Therefore, this study aims to innovatively integrate the BOPPPS instructional model with high-fidelity scenario-based simulation, designing and implementing a specialized training program for junior operating room nurses focusing on circulating duties. The research will systematically evaluate the effectiveness of this program in enhancing participants’ theoretical knowledge, clinical operational skills, emergency response capabilities, and professional self-efficacy. Furthermore, it will observe improvements in their work quality and nursing competency within real clinical environments. This study not only seeks to provide empirical evidence and a practical framework for optimizing the clinical competency development pathway for junior operating room nurses but also attempts to offer novel methodological references and practical insights for conducting structured, scenario-based in-service education and training in highly complex clinical settings such as the operating room.

## Participations and methods

### Research participants

Using purposive sampling, a total of 20 junior nurses (N2 level) who had completed all surgical rotations and possessed 3–5 years of work experience were selected from the operating room as study participants. The participants were then randomly divided into four groups of five each by means of a random number table.

### Theoretical framework

This study employs the Jeffries Simulation Theory as its theoretical foundation. Its core elements—Background, Design, Simulation Experience, Assessment, and Debriefing—can be systematically aligned with the six modules of the BOPPPS model, forming a reinforced closed loop of “Objective-Simulation-Reflection.” Specifically: (1) The Bridge-in and Objectives phase corresponds to the “Background and Design” components of Jeffries’ theory, utilizing authentic clinical cases to introduce problems and establish specific, observable learning objectives, thereby setting a clear direction for the simulation. (2) The Pre-assessment stage refines the “Design” element by assessing learners’ baseline knowledge and skills, allowing for personalized adjustment of simulation difficulty. (3) The core of Participatory Learning is embodied in Jeffries’ “Simulation Experience,” where learners engage in decision-making and operations within highly realistic, risk-free surgical scenarios. (4) The Post-assessment and Summary phases strictly align with “Assessment and Debriefing,” employing structured evaluation tools (e.g., checklists) and facilitated debriefing to transform the simulation experience into profound reflection and transferable conceptual understanding [11, 12].

### Research methods

This study employed a self-controlled quasi-experimental design rather than a randomized controlled trial (RCT) design. The rationale for this approach is twofold. First, all 20 participants were recruited from a single center, which would increase the risk of inter-group contamination if parallel-group comparisons were conducted. Second, the 20 nurses represented the entire population of eligible junior circulating nurses at the center, rendering the sample size insufficient to support parallel-group randomization. Therefore, a self-controlled quasi-experimental design was adopted to address these methodological constraints, incorporating the BOPPPS training approach, which comprises bridge-in, objective, pre-assessment, participatory learning, post-assessment, and summary for educational intervention. The implementation involved six sequential steps: bridge-in (B), objective (O), pre-assessment (P), participatory learning (P), post-assessment (P), and summary (S).

#### Bridge-in (B)

1. Problem screening
  Work-related video recordings relevant to the research participants were selected for review. In collaboration with the head nurse, the teaching group leader, and the quality control group leader, work quality was assessed using a self-developed *Circulating Nurse Work Content Execution Verification Checklist* (Appendix 1). Based on the assessment results, key issues in circulating nursing practice were identified for targeted intervention.
2. Cause analysis
  A combined session involving brainstorming, focused interviews, and root cause analysis was conducted. Participants included the head nurse, the teaching group leader, the quality control group leader, teaching faculty, relevant staff members, and peer representatives. The root causes of the identified issues were discussed and analyzed in detail and the specific findings are presented in Table 1.
3. Determine the root causes
  The head nurse, teaching group leader, quality control group leader, teaching staff, involved personnel, and peer representatives assessed the identified contributing factors by assigning importance scores ranging from 1 to 5. These factors were then ranked in descending order according to the assigned scores. Root causes were determined using the 80/20 principle. The primary causes identified were as follows:① Overlapping responsibilities and limited overall planning ability; ② Inefficient work paths and suboptimal time management; ③ Limited clinical reasoning skills; ④ Absence of a structured workflow for key circulating nurse responsibilities; ⑤ Inadequate awareness of patient safety measures; ⑥ Poor collaboration in anesthesia-related tasks and underdeveloped teamwork skills;⑦ Deficient communication skills and techniques; ⑧ Incomplete mastery of relevant professional knowledge and competencies.

**Table 1:** Root cause analysis

Problems to be addressed	Cause analysis
Knowledge and skills: Unfamiliarity with positioning methods and procedures; incomplete preparation of positioning items; incorrect procedures in positioning and facility use; insufficient knowledge regarding pre-warming; inappropriate handling of sterile items (e.g., wearing gloves).	Incomplete mastery of professional knowledge and skills.
Clinical thinking: Preoperative patient assessment and intraoperative condition observation are not comprehensive, and nursing problems and solutions are not accurately identified.	Lack of clinical thinking ability
Communication skills: Inappropriate attitudes and methods of communication with patients, surgeons, and anesthesiologists during intraoperative nursing coordination.	1. Lack of communication knowledge and skills. 2. Insufficient awareness of the importance of communication.
Team collaboration: Failure to prepare required items promptly during anesthesia; lack of timely proactive nursing cooperation.	1. Lack of emphasis on anesthesia coordination work. 2. Poor teamwork ability.
Organization and coordination: Delayed patient restraint; inadequate implementation of privacy protection; tripartite verification not performed according to departmental regulations; intraoperative verification methods incorrect; unreasonable arrangement of circulating work pathways.	1. Inadequate awareness of patient safety protection. 2. Overlapping tasks and weak overall planning skills. 3. Inefficient work pathways and time management.

#### Learning objectives and plan (O)

**Fig. 1:**
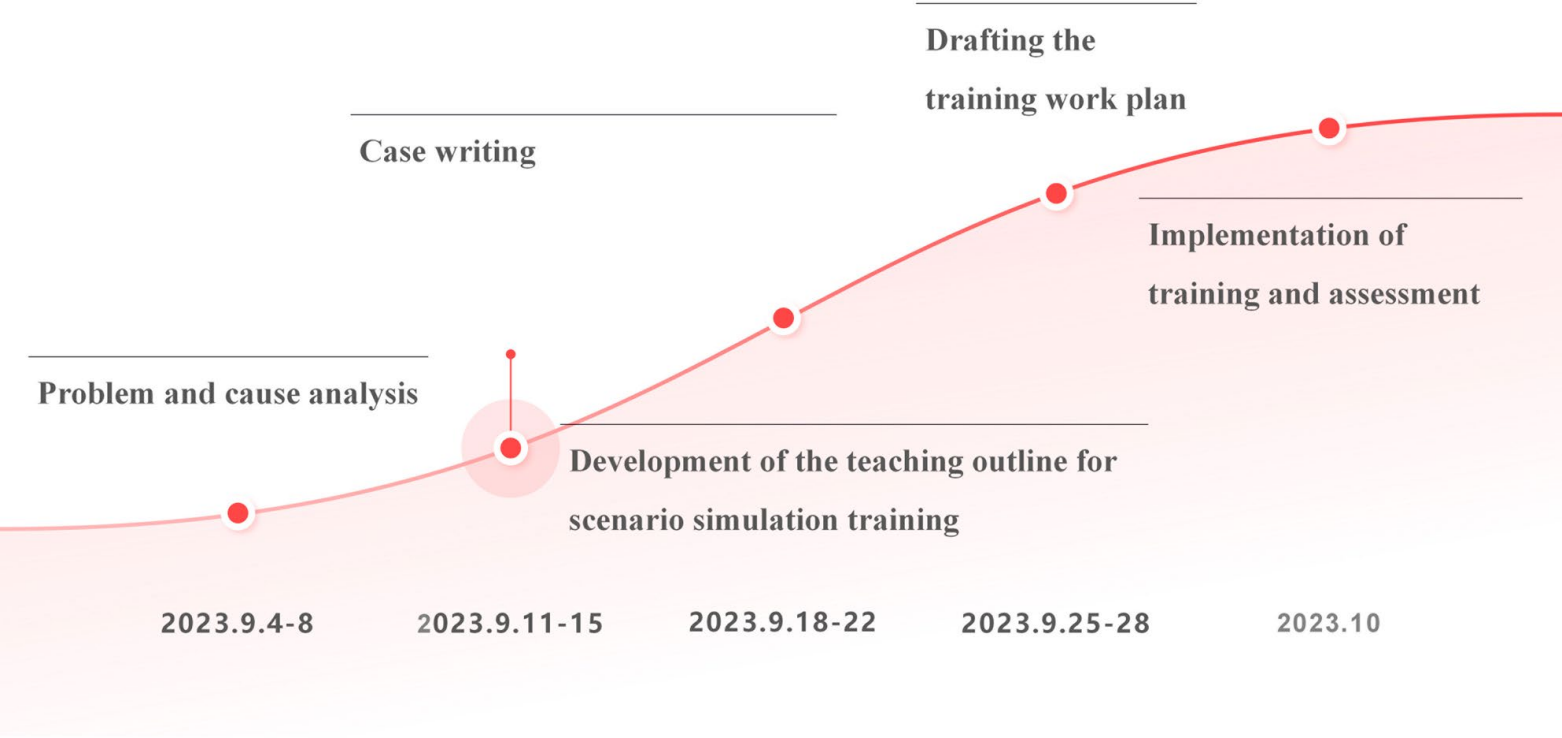
Training plan

1. Learning objectives
  1. Overall goal
    The overall objective was to enhance the quality of circulating nursing practice among junior nurses in the operating room. A minimum increase of ≥10% in the qualified rate and a ≥10% increase in the system execution rate were set as targets. These improvements were calculated using the following formula: target value = expected value (lower limit) + difference / difference * [1 - (current value - lower limit) / (upper limit - lower limit)].
  2. Breakdown of goals
    The following specific objectives were established: ① Enhancement of overall planning ability; ② Strengthening of clinical reasoning skills; ③ Development of self-directed workflows for key circulating nurse responsibilities; ④ Improvement of awareness regarding patient safety measures; ⑤ Strengthening of collaboration with anesthesia teams and promotion of teamwork;⑥ Enhancement of professional knowledge, technical skills, and communication competencies.
2. Training plan
  The training was conducted from September 2023 to October 2023. Detailed scheduling and content are presented in Figure 1.

#### Pre-assessment (P)

Preliminary data were collected through the review of video recordings. The evaluation of work quality included the following components:

1. Assessment of circulating work ability
  The *Comprehensive Ability Evaluation Form for Circulating Nurses* (Appendix 2), a self-developed tool, was used to assess the circulating work ability of 20 junior circulating nurses. The evaluation was jointly conducted by the head nurse, the teaching group leader, and the quality control group leader, while two instructors documented the assessment results.
2. Assessment of circulating work execution rate
  The *Circulating Nurse Work Content Execution Verification Checklist* (Appendix 1), a self-developed instrument, was used to assess the work execution performance of 20 junior circulating nurses. The evaluation was jointly carried out by the head nurse, the teaching group leader, and the quality control group leader, while two instructors recorded the assessment outcomes.
3. Entrustable professional activities assessment
  The *Entrustable Professional Activities (EPA) Evaluation Form for Operating Room Nurses’ Circulating Work* (Appendix 3), a self-developed tool, was used to assess the level of entrustability among 20 junior circulating nurses. Assessment results indicated that all 20 nurses achieved a score of 4 points, reflecting the ability to independently perform circulating duties.

#### Participatory learning (P)

Scenario-based simulation training was implemented in a real operating room setting. A total of 20 junior nurses participated in simulation exercises focused on circulating work tasks. The specific implementation process was as follows:

1. Developing scenario-based simulation cases
  Simulation cases were developed based on common issues identified in pre-training operating room video recordings, combined with the five lowest-scoring items from the ability assessments. Following joint discussions and iterative revisions conducted by the head nurse, members of the quality control group, and members of the teaching group, a final set of five scenario-based teaching cases was finalized (Appendix 3).
2. Implementation of scenario simulation
  Implementation of Scenario Simulation: On the day of training, four simulation scenarios were randomly selected from a pool of five cases for instructional implementation. In each group, four nurses took turns assuming the roles of scrub nurse, circulating nurse, surgeon, and anesthesiologist. During the simulation, the head nurse, teaching team leader, and quality control team leader observed the session via the operating room video monitoring system. Additionally, two instructors were present on-site as designated observers to document the process.
3. Guided Debriefing in Clinical Simulation: Following the completion of a scenario-based simulation, the debriefing process begins with self-assessment and peer review among the participating nurses. During this phase, they identify issues encountered during the simulation and analyze the key and challenging knowledge points involved. Subsequently, the head nurse, the teaching team leader, and the quality control team leader provide summative evaluations and guided feedback. The entire session is documented in real-time by two teaching faculty members.

For instance, in a scenario where safety restraints were not applied during surgical positioning, a structured guided debriefing protocol should be implemented:

First, the circulating nurse involved is asked to explain the reasoning behind their actions.

Next, fellow nurses are encouraged to share insights from clinical practice, discussing the importance and necessity of intraoperative restraints in preventing patient displacement, falls, and medical device dislodgement, thereby fostering a shared team understanding.

Finally, the head nurse summarizes the discussion, clarifies the standard operating procedures and evidence-based rationale for applying restraints, systematically reviews the standard workflow for patient positioning, and uses this opportunity to reinforce the core principles of patient safety. This approach helps junior nurses develop risk awareness, achieving the systematic educational objectives of progressing from individual case reflection to internalization of standards, and from corrective action to the cultivation of a safety culture.

#### Post-assessment (P)

On the day following the training, the quality and satisfaction related to circulating nursing work were assessed by the head nurse, the teaching group leader, and the quality control group leader through review of the recorded training video. ① Quality Evaluation of Work: Conducted using the same methods and tools as in the pre-assessment. ② Satisfaction Evaluation: The *Satisfaction Evaluation Form for Operating Room Circulating Nurse Scenario Simulation Training Course* (Appendix 4) was used to assess the satisfaction levels of 20 junior nurses regarding the scenario-based simulation training course and its perceived effectiveness.

#### Summary (S)

Following the training, a summary and evaluation were conducted by the head nurse, the teaching group leader, and the quality control group leader. The process included the following components: ① Individual Participation in the Review: Issues encountered during circulating work were jointly discussed by the participating nurses and their peers, with practical solutions proposed through collaborative review. ② Effect Feedback Form: Observations were documented on-site by instructors from the teaching building. Review content was compiled into a training effect feedback form and distributed to individual participants. ③ Individual Mind Map of Circulating Nursing Workflow: Trainees were instructed to outline their personal workflow processes and develop a corresponding mind map for future reference. ④ Confirmation and Feedback Submission: Each trainee confirmed the contents of the feedback form and submitted a written summary of their corrective strategies to the teaching group.

### Evaluation indicators

The effectiveness of circulating work training for junior nurses was assessed using the Kirkpatrick Four-Level Evaluation Model. This model comprises four hierarchical levels, which were used to establish the evaluation indicators for the present study [9].

#### Comprehensive ability evaluation form for Circulating nurses

The evaluation form (Appendix 2) was structured into five components: evaluation dimensions, evaluation content, scoring, evaluation standards, and evaluation methods. The content covered both the basic workflow and specific task requirements essential to the role of circulating nurses. A total of 7 dimensions and 20 items were included, encompassing operating room preparation, patient transportation, safety checks, intraoperative safety management, surgical nursing cooperation, documentation, and postoperative room organization. Each item was assigned a maximum score of 5 points. The total score was calculated out of 100, with 60 points designated as the passing threshold. Scores from 61 to 80 indicated average performance, 81 to 95 reflected good performance, and scores of 96 and above were classified as excellent.This scale was assessed by an observer.

#### Circulating nurse work content execution verification checklist

The checklist (Appendix 1) consisted of 30 critical components and specific tasks related to circulating nurse responsibilities. Each item was assessed using three response options: “Yes,” “No,” and “Not Applicable.” The execution rate was calculated using the following formula: Execution Rate (%) = 1 − “No” (%).This checklist was assessed by an observer.

#### Satisfaction evaluation form for scenario simulation teaching training course

Evaluation Questionnaire: The satisfaction questionnaire comprised 15 items covering the following dimensions: course design, course objectives, course content, course schedule, training methods, assessment methods, evaluation criteria, guided feedback, and training effectiveness. Responses were rated on a 5-point Likert scale: 1 = very dissatisfied, 2 = dissatisfied, 3 = neutral, 4 = satisfied, 5 = very satisfied. The total possible score was 75 points.This satisfaction questionnaire was self‑rated by the study participants.

The aforementioned three checklists serve as objective observational assessment tools and do not constitute psychological measurement scales; thus, conventional validation of reliability and validity is not required. However, prior to implementation, the content validity of these checklists was evaluated through consultation with clinical instructors, administrative personnel, and research nurses within our institution. All items achieved mean scores above 4 points, with coefficients of variation ranging from 11% to 17%, confirming the feasibility of the content and demonstrating strong consensus among expert evaluators.

#### Number of adverse events

The number of adverse events occurring during circulating nursing work among the 20 nurses was compared between September and October 2023. The classification of adverse events was based on the *Quality Management Indicators* outlined in the *Chinese Nursing Practice Guidelines*. Eight indicators related to circulating nursing responsibilities were included: ① Surgical safety checks; ② Surgical site identification; ③ Timing of antibiotic administration; ④ Acquired pressure injuries during stage II surgery; ⑤ Intraoperative hypothermia; ⑥ Intraoperative low-temperature burns; ⑦ Falling out of bed/falls.

### Statistical analysis

Statistical analyses were performed using SPSS 19.0. Continuous data are presented as mean±standard deviation (SD). Paired t-tests were used to compare pre- and post-training scores for work quality and work completion rate, as these variables were continuous and paired measurements were available. The number of adverse events is reported as a count. Satisfaction and work completion rates are expressed as percentages. The association between work quality and completion rate was assessed using Pearson correlation analysis. A two-sided p-value < 0.05 was considered statistically significant.

## Results

### Comparison of work quality scores prior to and following training

A comparison of work quality scores before and after training was conducted. As the data were normally distributed, a paired-sample t-test was performed. The results indicated that the post-training work quality score (85.05 ± 3.63) was significantly higher than the pre-training score (71.6±0.37), The magnitude of improvement was 18.78%,with *p* < .001 (Table 2).

**Table 2:** Comparison of work quality scores before and after training (*N* = 20)

Groups	Sample size	Mean value	t value	*P* value
Before training	20	71.6 ± 6.37	34.52	0.00 (*p* < .001)
After training	20	85.05 ± 3.63		

### Number of adverse events

The incidence of adverse events among the 20 junior nurses was tracked in September and October 2023. The number of pressure injury cases decreased from 2 to 0, while the occurrence of intraoperative hypothermia in surgical patients was reduced from 30 cases to 8 cases, corresponding to a decline in its incidence rate from 1.82% to 0.53%. (Table 3).

**Table 3:** Number of adverse events

Items	Surgical Volume in September	Number of cases in September	Surgical Volume in October	Number of cases in October
Safety checks	1645	0	1518	0
Surgical site identification		0		0
Timing of antibiotic administration		0		0
Acquired pressure injuries during stage 2 surgery		2		0
Intraoperative hypothermia		30		8
Intraoperative low temperature burns		0		0
Falling out of bed/falls		0		0

### Comparison of work execution rates prior to and following training

Since the data were normally distributed, a paired-sample t-test was employed for the comparison. The post-training completion rate (82.55%) was significantly higher than the pre-training rate (69.95%) ,*p* < .001 (Table 4) .

**Table 4:** Comparison of work execution rates before and after training (*N* = 20)

Groups	Sample size	Execution rates (mean value)	t value	*P* value
Before training	20	69.95%	32.30	0.00 (*p* < .001)
After training	20	82.55%		

### Training satisfaction

Overall satisfaction with the training was 96.11%. Satisfaction scores across individual dimensions are presented in Table 5.

**Table 5:** Satisfaction of various dimensions.(*N* = 20)

Indicators	Score	Satisfaction	Ranking
Course setup	75	100%	1
Course objectives	66	88%	3
Course content	75	100%	1
Course arrangement	75	100%	1
Training methods	75	100%	1
Assessment methods	68	90%	2
Evaluation standards	65	87%	4
Guiding feedback	75	100%	1
Training effectiveness	75	100%	1

### Data on process

#### The time allocation for each phase of the BOPPPS teaching model

In a specialized training program for junior circulating nurses in the operating room, the time allocation for each phase of the BOPPPS teaching model concretely and explicitly reflects its systematic and diagnostic instructional design logic.

1. The study began with an intensive 5-day (September 4-8) video observation period, which concurrently accomplished the core tasks of the "Bridge-in" and "Pre-assessment" phases. By analyzing work recordings of the 20 participants, common and individual issues in their clinical practice were accurately "diagnosed," while a baseline assessment of their competencies was simultaneously completed. This design efficiently integrated "problem identification" and "baseline evaluation," establishing an evidence-based foundation for subsequent targeted instruction.
2. Subsequently, a substantial 10-day period (September 11-22) was dedicated to the "Objectives" phase, specifically for developing the simulation training syllabus and four teaching cases. This step profoundly underscores a critical prerequisite for the integrated model's success: translating clear, measurable teaching objectives into highly realistic, well-structured simulation scenarios that closely align with clinical realities. This approach moves beyond merely applying existing cases, ensuring a high degree of consistency between simulation content and training goals.
3. The most crucial phases—"Participatory Learning," "Post-assessment," and "Summary"—were integrated into a cyclical format conducted over four full weeks in October (totaling 20 days). Each week, a group of five nurses completed the entire closed loop, from immersive scenario simulation and immediate skills post-testing to structured, guided debriefing. Within the integration of BOPPPS and simulation, the immediate assessment and in-depth debriefing following "Participatory Learning" constitute the most critical link for fostering competency internalization and must be allocated sufficient and structurally safeguarded time.

#### Level of learner engagement

In the present training program for junior circulating nurses in the operating room, the “Participatory Learning” phase achieved an exceptionally high level of learner engagement. Specifically, all 20 participants demonstrated 100% attendance and active participation rate. Each participant completed all four instructional cases and, through a mandated rotation across five distinct role-specific tasks within each case, achieved comprehensive immersion and in-depth experience within the simulated scenarios. The integrated BOPPPS and scenario-based simulation model effectively enhances learning engagement and comprehensive competency. The observed 100% participation rate and the multi-role rotation mechanism in this practice precisely reveal, at an operational level, the key design logic for achieving such high effectiveness: it systematically breaks learners’ fixed perspectives through mandated role rotation. This approach ensures that participants not only practice their own role-specific skills but also gain a profound understanding of the responsibilities, decision-making pressures, and collaborative needs of other team members (such as scrub nurses and anesthesiologists). Thereby, through “doing” and “experiencing,” they construct a systemic cognition of the entire surgical context.

## Discussion

This study designed and implemented a specialized training course for junior circulating nurses in the operating room by systematically integrating the BOPPPS teaching model with high-fidelity scenario simulation. The results demonstrated that this integrated teaching approach effectively improved the quality of circulating duties and core compliance rates, while also achieving high training satisfaction. The following discussion will elaborate on the potential mechanisms underlying these findings, their relationship with existing literature, and the innovative contributions of this research.

Synergistic Improvement in Circulating Work Quality and Compliance: Translating Knowledge into Integrated Competency. Following the training, the score for circulating work quality significantly increased from 71.6 ± 6.37 to 85.05 ± 3.63, while the job compliance rate improved from 69.95% to 82.55%. This finding aligns with the conclusion by Tian et al. [13] that simulation training can enhance nurses’ job competency. However, by employing the structured BOPPPS framework, this study further systematized and made this transformation process more explicit.

The enhancement in work quality stems from the multi-reinforcement effect of the training design. Firstly, during the “Pre-assessment” and “Objective” phases of the BOPPPS model, competency gaps and learning objectives were clearly defined, thereby making the subsequent scenario simulation (“Participatory Learning”) more targeted [14]. Secondly, the structured feedback (particularly the three-tiered guided feedback) provided in the “Post-assessment” and “Summary” phases prompted deep metacognition among the nurses, helping them identify deficiencies in their judgment and actions during the simulation. This process facilitated the crucial transition from merely “experiencing an event” to “internalizing competency.” Consequently, the improvement in the compliance rate reflects not merely increased behavioral adherence but also an external manifestation of enhanced self-confidence (self-efficacy) and clinical judgment among the nurses. This aligns with the observation by Xie et al. [15] that lack of confidence is a key factor affecting work quality and adverse patient outcomes. The intervention in this study directly addressed the root causes of low self-efficacy [16] and poor compliance [17] among junior nurses, which often stem from insufficient knowledge and skills compounded by high-pressure environments. By offering a safe and supportive simulation training experience, the intervention helped break the vicious cycle of “inadequate competency → low confidence → poor performance.”

Based on Jeffries’ Simulation Theory, the mechanism through which the integrated BOPPPS and high-fidelity simulation intervention in this study promotes clinical competence follows a closed-loop pathway of “contextual activation → structured reflection → cognitive reconstruction.” This theory emphasizes that effective teaching relies on the interaction among “design characteristics,” “simulation practice,” and “educational practices” (with guided debriefing as the core). The intervention first activates learners’ implicit mental models through high-fidelity simulation scenarios, exposing the “performance gap” between their cognition and actions when confronting complex tasks. The critical transformation occurs during the “Post-assessment and Summary” phase of BOPPPS, i.e., structured guided debriefing. Here, facilitators employ behavior-evidence-based questioning to prompt learners to systematically compare their decisions with clinical standards. The resulting cognitive conflict serves as the core driver of learning. To resolve this conflict, learners, under guidance, deconstruct the maladaptive components of their original cognition and integrate contextual experience with theoretical knowledge, thereby constructing a more accurate and adaptive new cognitive-behavioral model. Ultimately, this newly reshaped cognitive structure, forged through the “experience-reflection” closed loop, is consolidated via memory reconsolidation mechanisms. It becomes an internal foundation that can be rapidly and automatically retrieved and applied in future real clinical situations, achieving a fundamental transition from declarative knowledge to contextualized competence.

While existing studies have respectively confirmed the structural advantages of the BOPPPS teaching model in ensuring the achievement of teaching objectives and process systematization [9, 14, 18], as well as the general efficacy of scenario-based simulation in promoting skill transfer and enhancing on-the-spot decision-making ability [17], most empirical evidence remains grounded in conventional, standardized applications of simulation-based pedagogy. Although such research validates the general effectiveness of these methods, it often fails to adequately address the unique challenges and the deeper training objectives inherent in specific high-risk, high-complexity clinical environments.

Therefore, the core academic value of this study lies not in introducing a novel teaching methodology, but precisely in the deep integration and systematic application of these two established and validated methods—BOPPPS and scenario-based simulation—to the quintessential high-stakes setting of the operating room. Its contribution resides in employing rigorous empirical investigation to explore and verify whether this integrated model can transcend foundational skill training, thereby addressing the critical challenge of cultivating advanced, composite competencies in operating room nursing. These include dynamic situational judgment, multi-threaded task management, team collaboration under pressure, and risk anticipation.

Provision of a Comprehensive Theoretical Framework from Training Design to Evaluation.

This study did not merely incorporate simulation cases into a traditional curriculum; instead, it systematically structured the entire simulation-based instruction using the six-phase BOPPPS model. From creating urgent clinical scenarios in the “Bridge-in” phase to stimulate learning motivation, to distilling simulation experiences into transferable work principles during the “Summary” phase, this approach ensured that each simulation constituted a complete, goal-driven learning loop. This design addresses the shortcomings observed in some previous simulation training, which may suffer from loose design, ambiguous objectives [18, 19], and an over-reliance on subjective psychological evaluation [1, 9, 11].

Achieving a Dynamic Balance Between “Targeted Training” and “Immersive Experience”.

The “Pre-assessment” phase facilitated the identification of both common and individual weaknesses among the trainees, allowing for dynamic adjustment of the difficulty level and focus of the simulation scenarios to achieve personalized instruction. Concurrently, allocating 80% of the training time to high-fidelity simulation practice ensured sufficient immersive experience. This enabled learners to apply their knowledge under highly realistic pressure, which is more effective than traditional lecturing or isolated skill drills in cultivating the comprehensive adaptive competence required in actual operating room settings.

Enhancing the Systematic and Formative Nature of Feedback.

The built-in “Post-assessment” and “Summary” phases of the BOPPPS model necessitate that feedback be grounded in concrete, observable performances (e.g., comparison of pre- and post-assessment data, critical incidents during simulation). This structure elevates the “feedback-discussion-improvement” cycle beyond subjective description to a level of evidence-based reflection, effectively promoting the consolidation and advancement of competency [20].

Insights from Training Satisfaction and Pathways for Continuous Optimization.

A satisfaction rate of 100% regarding course design, content, and feedback methods validates the advantages of the integrated model in terms of engagement and learner participation. However, the relatively lower satisfaction scores for “course objectives” (88%) and “assessment criteria” (87%) precisely reveal a critical component in structured teaching: consensus on goals. This finding aligns with the ADDIE model’s emphasis on the paramount importance of the analysis and design phases [21]. It suggests that in future training implementations, more time must be invested in the “Objective” phase. Utilizing methods such as pre-reading materials and group discussions to ensure learners fully understand and internalize the learning objectives and evaluation dimensions can thereby stimulate stronger intrinsic motivation. This approach is consistent with the concept of enhancing job competency by integrating Entrustable Professional Activities [22].

Advancing the clinical teaching quality in the operating room:

Guided by the BOPPPS framework, this study constructed a structured, participatory six-stage teaching cycle (Bridge-in, Objective, Pre-assessment, Participatory Learning, Post-assessment, and Summary). Its core innovation lies in the deep integration of scenario-based simulation, which employs authentic clinical case designs, multi-role immersive drills, and a tri-level guided feedback system (self-, peer-, and instructor-led) to facilitate the translation of knowledge into practical competence. A multidimensional evaluation system was also established, integrating competency assessments, checklist compliance rates, satisfaction surveys, and adverse event tracking to quantify training outcomes. Based on these findings, recommendations for future clinical teaching in the operating room are proposed: dynamically refining training focus and clarifying objectives using pre-assessment data, enhancing the instructional team’s capabilities in scenario facilitation and feedback delivery, and cultivating a sustainable culture of teaching quality—thereby continuously improving nurses’ job competence and ensuring patient safety.

Although the integrated BOPPPS and simulation model holds significant theoretical advantages in its design, its successful application in practice is not guaranteed and may fail to achieve expected outcomes for various reasons. It also does not fully overcome the inherent limitations of simulation-based teaching itself. The integrated model faces dual challenges in practical application that may constrain its intended effectiveness. Firstly, it demands exceptionally high execution fidelity: inaccurate pre-assessment or debriefing sessions that fail to provoke deep reflection can result in learning remaining at the level of superficial practice; furthermore, mechanically applying the instructional framework can undermine the immersive quality of the simulation. Secondly, it does not entirely surmount the intrinsic constraints of simulation pedagogy: the transfer of learning outcomes to real, high-pressure surgical environments remains uncertain; concurrently, the high costs associated with equipment, case development, and instructor training hinder its widespread adoption; additionally, the evaluation of non-technical skills lacks objective criteria. Therefore, its success depends not only on rigorous design but, more critically, on comprehensive instructor training, sustained resource support, and empirical tracking of long-term skill transfer.

### Limitations

This study possesses several limitations that warrant careful consideration. First, the adoption of a single-center, small-sample, pre-post design without a parallel control group represents a significant methodological constraint. This design flaw may substantially overstate the perceived intervention effect. Due to the inability to control for confounding variables—such as historical events, maturation effects (e.g., natural skill progression with work experience), or concurrent training activities—the observed performance improvements cannot be robustly and solely attributed to the intervention, thereby weakening the strength of causal inference.

Second, the study relied on self-developed clinical behavior observation checklists as the primary assessment tool, without established reliability and validity. This directly threatens the study’s internal validity. The unverified measurement reliability (stability and consistency) of these tools may be insufficient, potentially leading to inter-rater discrepancies or random error. More critically, their construct validity is questionable—specifically, whether the tools accurately and unbiasedly measure the intended constructs (e.g., “circulating competency”) rather than confounding irrelevant traits (e.g., performance enthusiasm or familiarity with the assessor). This limitation constrains the authenticity and comparability of the data, challenging the reliability of the conclusions.

Finally, all assessors were affiliated with the same research center. While familiarity with participants might enhance contextual understanding during observation, it could also compromise the objectivity and independence of assessments due to unconscious expectancy bias or social desirability pressure.

Collectively, these limitations indicate that the current positive findings should be regarded as preliminary evidence. The certainty of the conclusions, their generalizability, and the estimation of effect sizes require confirmation and further substantiation through future research employing randomized controlled designs, standardized or rigorously validated assessment tools, and independent blinded assessors.

## Conclusion

This study demonstrates that integrating the structured BOPPPS instructional model with high-fidelity scenario simulation can systematically and significantly enhance the circulating work quality and task completion rates of novice operating room nurses. Its effectiveness is empirically supported by quantified performance improvements, such as a marked increase in work quality scores. This core finding provides a crucial implication for educational practice: in clinical skills training, the deep integration of rigorous instructional design (exemplified by the phased, closed-loop structure of BOPPPS) and immersive practice (simulation) constitutes a highly efficient pathway for translating theoretical knowledge into reliable clinical performance.

Based on study feedback, optimizing practice should focus on three key directions: goal precision (clearly articulating objectives and evaluation criteria prior to training), process personalization (dynamically adjusting scenario complexity based on pre-assessment results), and systematic assessment (employing structured frameworks such as Kirkpatrick’s Four-Level Evaluation Model to conduct long-term tracking across reaction, learning, behavior, and result levels). This suggests that future pedagogical innovation lies not merely in methodological integration but, more importantly, in establishing a closed-loop, evidence-based system of “diagnosis-training-assessment-improvement.”

For subsequent research, it is essential to employ systematic evaluation frameworks (e.g., the Kirkpatrick model) to thoroughly measure the long-term transfer effects of skills to real clinical environments and their organizational impact. Furthermore, exploring the integration of technologies such as virtual reality may help overcome bottlenecks in cultivating higher-order competencies. These efforts will provide robust evidence for constructing a scientific, evaluable, and sustainable paradigm for developing specialist nurse competency.

## Supplementary Information

Supplementary Material 1

## Data Availability

The datasets used and/or analysed during the current study available from the corresponding author on reasonable request.

## Abbreviations

BOPPPS: (bridge-in, objective, pre-assessment, participatory learning, post-assessment, summary)
