# Supplementary material for: Evaluating the impact of a BOPPPS and scenario-based simulation intervention on the competency of junior circulating nurses: a quasi-experimental study
Source: BMC Med Educ. 2026 Feb 2;26:368. doi: 10.1186/s12909-026-08712-y (PMC12955285; doi:10.1186/s12909-026-08712-y)
Supplement: Supplementary file 1 — Supplementary Material 1 [file 12909_2026_8712_MOESM1_ESM.docx]

Appendix 1: Circulating Nurse Work Content Execution Verification Checklist

| Work content | Yes | No | Not applicable |
| --- | --- | --- | --- |
| Patient evaluation: Assessment of patient condition and overall status |  |  |  |
| Evaluation of the operating room: Laminar flow, anesthetic waste gas, temperature, closing of front and back doors, presence of gauze in trash bins, items left from previous surgery affecting inventory, validity of disinfectant, pathology box, incubator, refrigerator. |  |  |  |
| Item preparation: Positioning materials, instruments, and equipment |  |  |  |
| Correct method of equipment application |  |  |  |
| Verification upon patient entry: Information, surgical site identification, infection screening, items, and medications |  |  |  |
| Is there safety protection and privacy protection for patients? |  |  |  |
| Tri-party verification: Personnel, timing, and content performed according to protocol. |  |  |  |
| Whether to participate in and cooperate with anesthesia? |  |  |  |
| Proper preparation of antibiotics. |  |  |  |
| Is the positioning carried out according to the correct principles and procedures? |  |  |  |
| Pay attention to the scope of surgical disinfection and the usage of disinfectants. |  |  |  |
| Is the inventory count conducted according to department regulations and principles? |  |  |  |
| Preparation and correct use of consumables (high- and low-value), including verification of brand, model, and date. |  |  |  |
| Accurate and complete documentation. |  |  |  |
| Timely and accurate charging. |  |  |  |
| Monitoring of patient vital signs and confirmation of unobstructed tubes during procedure |  |  |  |
| Attention to changes in body position and skin pressure during procedure, with regular release of pressure. |  |  |  |
| Whether to manage the personnel and environment during the procedure? |  |  |  |
| Correct handling of sterile items and consumables |  |  |  |
| Management of intraoperative emergencies (hemorrhage, cardiac arrest). |  |  |  |
| Monitoring of surgical progress and timely provision of necessary materials. |  |  |  |
| Surgical-pathological management: Proper preservation of specimens during and after surgery; accurate completion of pathology forms |  |  |  |
| Whether to provide protection to the patient after the end of surgery. |  |  |  |
| Pay timely attention to the skin condition after surgery (whether issues can be properly handled and forms are filled out correctly, and understanding the adverse event reporting process). |  |  |  |
| Confirmation that all patient belongings are accounted for before leaving the operating room. |  |  |  |
| If there is no scrub nurse, handle the instruments properly. |  |  |  |
| Postoperative operating room organization and checks: Beds, lights, laminar flow, anesthetic waste gas, suction devices, anesthetic drugs, equipment powered off and stored properly, positioning items returned and counted, pathology box, incubator, refrigerator. |  |  |  |
| With good communication skills |  |  |  |
| With teamwork skills |  |  |  |

Appendix 2: Comprehensive Ability Evaluation Form for Circulating Nurses

| Evaluation dimensions | Evaluation content | 1 point | 2 points | 3 points | 4 points | 5 points |
| --- | --- | --- | --- | --- | --- | --- |
| Operating room preparation | Verification of laminar flow, anesthetic waste gas, room temperature, closure of front and back doors, absence of gauze in trash bins, absence of leftover items that may affect the next surgical inventory, validity of disinfectant, and readiness of pathology box, incubator, and refrigerator. |  |  |  |  |  |
| Patient transport | Verification of patient information upon entering the operating room (personal information, surgical site id |  |  |  |  |  |
|  | Patient safety protection and privacy protection. |  |  |  |  |  |
| Safety verification | Timing (preoperative, intraoperative, postoperative), personnel (anesthetists, doctors, nurses). |  |  |  |  |  |
| Intraoperative safety management | Anesthesia coordination (materials, restraints, timing). |  |  |  |  |  |
|  | Positioning: joints, blood vessels, nerves, pressure injuries, pre-warming, hypothermia protection |  |  |  |  |  |
|  | Monitoring of surgical disinfection scope and appropriate use of disinfectants. |  |  |  |  |  |
|  | Observation of patient vital signs and confirmation of unobstructed tubes. |  |  |  |  |  |
|  | Pay attention to changes in body position and skin pressure during the procedure and release pressure regularly. |  |  |  |  |  |
|  | Management of personnel and environment in the operating room. |  |  |  |  |  |
| Surgical nursing cooperation | Inventory counting (timing, methods, and content) |  |  |  |  |  |
|  | Proper handling of sterile items and consumables |  |  |  |  |  |
|  | Advance preparation and correct use of high- and low-value consumables (including verification of brand, model, and expiration date) |  |  |  |  |  |
|  | Monitoring of surgical progress and timely provision of required materials |  |  |  |  |  |
|  | Appropriate use of equipment and basic troubleshooting. |  |  |  |  |  |
|  | Management of intraoperative emergencies (e.g., hemorrhage, cardiac arrest). |  |  |  |  |  |
|  | Surgical-pathological management (correct specimen preservation during and after surgery, and accurate completion of pathology forms). |  |  |  |  |  |
|  | If there is no scrub nurse, handle the instruments properly. |  |  |  |  |  |
| Document writing | Fill out the form correctly. |  |  |  |  |  |
| Postoperative organization | Operating room organization and checks: beds, lights, laminar flow, anesthetic waste gas, suction devices, anesthetic drugs, equipment powered off and returned to proper positions, positioning items counted and restored, pathology box, incubator, and refrigerator readiness. |  |  |  |  |  |

Instructions:

1. This form is scored using a checkmark (√). Based on the actual performance of the assessed nurse, the appropriate score (1–5) should be marked with a √. Any deficiencies should be documented in the explanatory section corresponding to the score.
2. Scoring criteria are defined as follows:

1 point: Most actions performed incorrectly; critical errors in sterile procedures; surgical progress adversely affected.

2 points: Some omissions present; most actions correct; no fundamental errors; surgical progress unaffected.

3 points: Meets basic requirements; generally acceptable performance with occasional omissions.

4 points: Qualified performance with no significant omissions.

5 points: Excellent performance; all actions completed accurately without omissions.

Appendix 3. Scenario Simulation Case

**Case 1. LS Whipple**

General Information: Name: ; Age: 35 years; Sex: Female; Height: 160 cm, Weight: 65 kg

Chief Complaint: Continuous upper abdominal pain for 6 months, accompanied by 20 kg weight loss, jaundice, and intermittent palpitations with shortness of breath.

History of present illness: The patient reported upper abdominal discomfort beginning six months prior, which progressively worsened. Symptoms were accompanied by jaundice, pruritus, dark urine, pale stools, and a weight loss of 20 kg. Three months earlier, intermittent palpitations and nocturnal shortness of breath developed. Electrocardiography revealed atrial fibrillation, leading to interatrial septal defect repair and pacemaker implantation. The patient was evaluated at several hospitals, where a pancreatic head tumor was suspected. For further management, she was admitted to undergoing laparoscopic Whipple surgery.

**Laboratory examination:**

Blood routine examination: Hb 80 g/L, erythrocyte hematocrit 0.25.

Tumor markers: CA199 750 U/ml (reference value < 37 U/ml).

Liver function: elevated transaminases, total bilirubin 160 umol/L (normal < 21 umol/L).

**Auxiliary examination:**

Abdominal contrast-enhanced magnetic resonance imaging (MRI) indicates a 5.5 × 6.5cm low-density mass in the pancreatic head with indistinct margins; mild enhancement after contrast; evidence of cholestasis due to pancreatic and bile duct compression. Cardiac ultrasound: Left atrial enlargement; post-intervention changes consistent with atrial septal defect repair and pacemaker placement.

**Diagnosis:**

Pancreatic head tumor with cholestasis

Non-rheumatic atrial fibrillation

Status post atrial septal defect repair and pacemaker implantation

Temporary medical orders: Cefotiam 3 mg brought to the operating room. Blood type: AB, Rh positive, prepare 2 units of red blood cells, 400 ml of plasma.

Before anesthesia:

Patient: I'm really so nervous and scared.

Frontline anesthetist: What are you afraid of? We do surgeries for patients like you every day.

**During the surgery:**

Surgeon: “The superior mesenteric vein is slightly torn. Anesthetist, please monitor blood pressure closely. Provide 5-0 Prolene sutures.”

Ten minutes later: Bleeding worsens, and blood pressure drops.

Anesthetist: “Quickly, obtain blood products.”

Surgeon: “Prepare for conversion to open surgery.”

**Case 2: LS Radical nephrectomy**

Male, 73 years old.

**Chief complaint:** Right flank pain for more than 3 weeks; right kidney mass identified on examination for 2 weeks.

**Current medical history:**

The patient developed intermittent dull pain in the right flank more than 3 weeks earlier, without an obvious cause. The pain was self-limiting and not associated with hematuria, fever, headache, dysuria, frequency, urgency, or painful urination. The patient initially attributed the symptoms to “kidney stones” and did not seek medical attention. Two weeks ago, as the pain worsened, abdominal ultrasound and computed tomography (CT) at a local hospital indicated a right renal mass. The patient was referred for further evaluation at a higher-level hospital. Nine days prior to admission, a contrast-enhanced upper and middle abdominal CT scan performed at this hospital demonstrated a nearly circular nodule (2.4 × 1.9 cm) protruding outward from the upper pole of the right kidney, suggestive of a tumor, although complicated cystic lesions could not be excluded. Clinical correlation was advised. The patient was admitted with a working diagnosis of “right kidney mass.” Since symptom onset, general condition, sleep, appetite, urination, and defecation remained normal, with no significant weight change.

**Past medical history:** No history of hepatitis, malaria, or tuberculosis. Hypertension for more than 2 years, with systolic pressure previously up to 160 mmHg. Currently treated with two unspecified antihypertensive agents, with blood pressure controlled at 130/82 mmHg. The patient reported fluctuations associated with anxiety and stress. History of femoral shaft fracture 3 years earlier, treated with open reduction and internal fixation.

LS right nephrectomy is scheduled today.

**Additional dialogue:**

Patient: “I brought the phone with me.”

Doctor: “No need to secure the leg straps, just tape it.”

Chief Resident: “When establishing the cavity, the balloon ruptured, leaving a 2 × 2 cm defect.”

**Case 3. TUR-BT**

The patient presented with total painless gross hematuria persisting for 26 days. Initial evaluation at a local hospital included urinary system CT, which revealed a mass in the left posterior wall of the bladder. He was subsequently admitted to the urology department of this hospital for further treatment, with a working diagnosis of bladder tumor. Since onset, general condition, sleep, and appetite remained stable, bowel habits were normal, and no significant weight changes were reported.

**Past medical history**

Hypertension, with a history of cardiac stent placement; Left hip replacement surgery five years prior; History of hepatitis B; Denies other comorbidities

**Planned surgery**
Transurethral resection of bladder tumor (TUR-BT) scheduled for the current admission, with transfer to the intensive care unit (ICU) planned postoperatively.

**Additional dialogue:**

Patient: “Do I have to take off my clothes? It’s really cold.”

Anesthetist: “Ask the circulating nurse for additional iodine.”

Doctor: “I’ll proceed without scrubs; it’s just a single small incision. After this, we need to add an emergency surgery.”

**Case 4: VATS Lobectomy**

**Chief complaint and history**

Patient: 53-year-old male

Chief complaint: Cough for 1 month, worsening for 2 days.

**Current medical history:** One month prior, the patient developed a cough attributed to a “cold.” No blood-stained sputum, dyspnea, or chest tightness was reported. Two days ago, the cough worsened following changes in weather. The patient sought evaluation at another hospital, where chest CT revealed a left lung mass. He was subsequently admitted for further diagnostic workup and treatment. Sleep quality has been reported as poor over the past three months.

**Past Medical History**: Diagnosed with ankylosing spondylitis in 1995; most recently managed with oral traditional Chinese medicine, discontinued one month earlier. Underwent right inguinal hernia repair in 2001.

Physical examination and auxiliary examination

The chest CT indicated a left lung mass.

**Diagnosis and management**

Diagnosis: Mass in the inferior lobe of left lung

Treatment Plan: Video-assisted thoracoscopic surgery (VATS) lobectomy of the inferior lobe of the left lung, with preparation for mediastinal lymph node biopsy

**Additional dialogue:**

Surgeon: “This wrist rest is too low—raise it, otherwise there is no place to stand.”

Scrub nurse: “Pulmonary lobe specimen is being sent for frozen section.”

Surgeon: “Arterial bleeding—anesthetist, monitor this. Is the blood pressure stable?”

Anesthetist (after a pause): “Cardiac arrest has occurred.”

**Case 5. Thyroid Surgery**

The patient noted a right-sided neck mass 3 months earlier. Neck ultrasound performed at a local hospital revealed a solid mass in the right lobe of the thyroid, described as a hypoechoic lesion with calcification, as well as bilateral cervical lymphadenopathy. The patient was admitted to the thyroid and breast surgery department of this hospital for further evaluation and treatment, with a working diagnosis of right lobe thyroid carcinoma. Since the onset of illness, the patient has reported depressed mood, reduced appetite, insomnia, and weight loss of approximately 2.5 kg compared with pre-admission status.

Past medical history: Anxiety disorder, diagnosed 2 years earlier, reported as cured; Cervical spondylosis for 5 years, treated with posterior open-door cervical laminoplasty; Allergy to adhesive tape

The patient is scheduled for radical thyroid cancer surgery and lymphadenectomy today.

**Additional dialogue:**

Patient: Repeatedly murmured concern regarding prognosis, stating poor sleep, anxiety, and fear, and asking how much longer life might be expected.

Anesthetist: Requested that the nurse assist with intravenous access.

Doctor: Instructed that the shoulder be raised and the head lowered further.

Scrub nurse: Reported abdominal discomfort to the instructor and requested permission to use the restroom.

**Appendix 4.** Satisfaction Evaluation Form for Operating Room Circulating Nurse Scenario Simulation Teaching Training Course

Instructions: Please evaluate each item by selecting the appropriate option.
Scale:
A. Very satisfied B. Satisfied C. Average D. Dissatisfied E. Very dissatisfied

1. How do you evaluate the course setup of this scenario simulation training?
2. How do you evaluate the course objectives of this scenario simulation training?
3. How do you evaluate the course content of this scenario simulation training?
4. How do you evaluate the course arrangement of this scenario simulation training?
5. How do you evaluate the training methods used in this scenario simulation training?
6. How do you evaluate the assessment methods of this scenario simulation training?
7. How do you evaluate the evaluation standards of this scenario simulation training?
8. How do you evaluate the guiding feedback provided in this scenario simulation training?
9. How do you evaluate the effectiveness of this scenario simulation training in terms of:
     (1) Stimulating learning interest
     (2) Improving the accuracy of work behaviors
     (3) Enhancing critical thinking
     (4) Supporting understanding of training content
     (5) Improving problem analysis skills
     (6) Reinforcing retention of key training points
10. How do you evaluate the attitudes of standardized participants (NPCs) in this scenario simulation?
11. How do you evaluate the realism of the scenarios and environment in this scenario simulation training?
12. How do you evaluate the teaching facilities used in this scenario simulation training?
13. How do you evaluate the overall implementation of this scenario simulation training?
14. How do you evaluate the training course as a whole?

Do you agree that this training h
